# Cultural differences in hydration practices among physically active individuals: a narrative review

**DOI:** 10.1080/15502783.2022.2057196

**Published:** 2022-04-04

**Authors:** Clarence Hong Wei Leow, Beverly Tan, Masashi Miyashita, Jason Kai Wei Lee

**Affiliations:** a Human Potential Translational Research Programme, Yong Loo Lin School of Medicine, 2 Medical Drive, National University of Singapore, Singapore; b Campus for Research Excellence and Technological Enterprise (CREATE), Singapore; c Faculty of Sport Sciences, Waseda University, Saitama, Japan; d Department of Physiology, Yong Loo Lin School of Medicine, National University of Singapore, Singapore; e N.1 Institute for Health, National University of Singapore, Singapore; f Global Asia Institute, National University of Singapore, Singapore; g Institute for Digital Medicine, Yong Loo Lin School of Medicine, National University of Singapore, Singapore; h Singapore Institute for Clinical Sciences, Agency for Science, Technology and Research (A*STAR), Singapore; i La Isla Network, Washington, District of Columbia, USA

**Keywords:** Culture, sports nutrition, hydration status, exercise performance, exercise recovery

## Abstract

It is well-established that appropriate hydration practices are essential in promoting health and optimizing performance and recovery. However, evidence-based hydration guidelines may not be adopted due to cultural differences across countries, such as religious beliefs, traditions, preferences, and beverage availability. Examples of hydration practices influenced by culture include beer consumption after sports in Western countries, consumption of sugarcane juice in India and Ramadan fasting among Muslims. For most cultural hydration practices, there is limited scientific evidence on their effects on rehydration, exercise performance, and recovery. Despite possible benefits of various hydration practices on exercise performance and recovery, they are inconsistent with current evidence-based hydration recommendations. More research on the impacts of cultural hydration differences on physiology, performance, and recovery is warranted to allow evidence-based guidelines and advisories.

**Abbreviations**: ABV: alcohol by volume, ACSM: American College of Sports Medicine, NATA: National Athletic Trainers’ Association, ROS: reactive oxygen species, TCM: Traditional Chinese Medicine

## 1. Background

Appropriate hydration practices are important for performance and health [1]. The National Athletic Trainers’ Association (NATA) and the American College of Sports Medicine (ACSM) provide guidelines on fluid replacement, for example, the types and amounts of fluids that should be consumed before, during and after exercise. These guidelines also advise against excessive sweat loss and exercise-associated hyponatremia [1,2]. Adverse consequences of improper hydration include both physical and physiological strain such as reduced performance and recovery [1,2]. Both the NATA and ACSM recommend an individualized hydration plan as fluid needs vary across individuals, depending on several extrinsic (e.g. exercise duration and intensity, environmental conditions) and intrinsic (e.g. heat acclimatization status, body mass, and size) factors [1,2]. Despite these guidelines, actual hydration practices might differ due to cultural differences among populations. Culture is defined as ‘the customs and beliefs, art, way of life and social organisation of a particular country or group’ [3]. As culture can influence diet, hydration practices can therefore be influenced by cultural reasons such as personal beliefs, beverage availability, and religion.

One cultural consideration is personal beliefs. For example, in Chinese culture, warm water is believed to be more beneficial for health than cold water [4]. Another reason is the availability of certain beverages in different parts of the world. Acai juice, sugarcane juice, and *beso* are often consumed in Brazil [5], India [6] and Ethiopia [7,8], respectively, because they are readily available in each country. In Western countries, beer is culturally preferred as it has become a part of the diet [9,10]. Religious beliefs can also create cultural differences in hydration practices. One prominent practice is Ramadan fasting in Islamic culture, where Muslims abstain from eating and drinking during daylight hours [11]. These cultural differences reveal that individuals might not necessarily adopt recommended hydration practices due to cultural influences.

**Figure 1.**
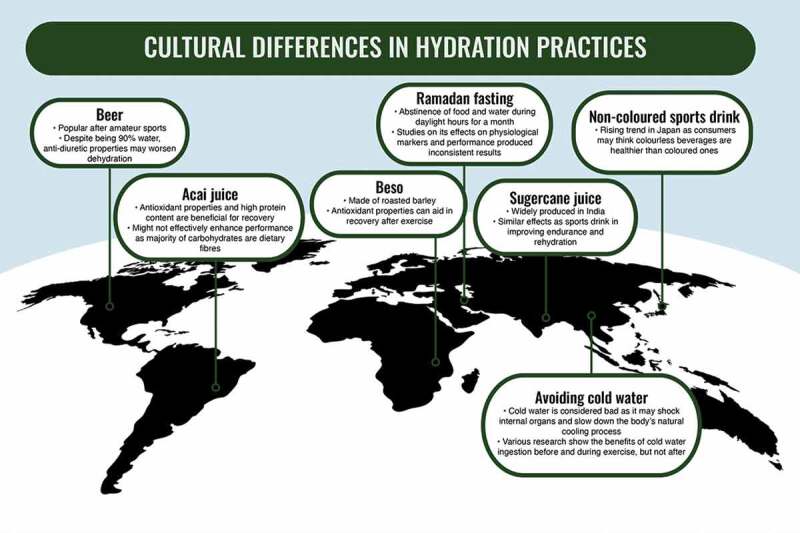
Summary of key observations for the various hydration practices around the world.

While most research focuses on the nutritional aspect of diets, the impacts of cultural differences have been not evaluated [12]. Therefore, we aimed to discuss existing cultural differences in hydration practices in the context of exercise among physically active individuals, specifically their potential impacts on rehydration, exercise performance and recovery (Figure 1). For this narrative review, a search of the published literature was performed on PubMed and Google Scholar using the terms “hydration”, “culture”, “exercise”, “performance”, and “recovery”. No restrictions were placed on language and date of publication of articles. Second- and third-order reference lists were also checked manually for relevant articles.

## 2. Cultural differences in hydration practices globally

### 2.1. Acai juice

Acai (*Euterpe oleracea*) is native to the Amazon region and Brazil is the major producer of acai [5,13–15]. Acai is prepared from its dark purple berries and usually consumed as a juice or smoothies. Acai began to grow in popularity among those who regularly engage in physical activity in the late 1990s [14]. Over the 21st century, acai has been well-promoted over the Internet. During the 2016 Summer Olympics in Rio de Janeiro, it received much publicity after various Olympians shared about the fruit on social media platforms [16].

Acai is commonly known as a ”superfood” as it is believed to confer a wide array of health benefits due to its antioxidant [13–15] and anti-inflammatory [13,14,17] properties. These properties are associated with the presence of polyphenols. The main polyphenolic compounds found in acai are anthocyanins, proanthocyanins, and other flavonoids [15]. Acai is also rich in essential fatty acids, dietary fiber, proteins, and minerals such as sodium and potassium [15].

While there is a lack of literature on the effect of consuming acai juice on hydration status, it is possible that there may be beneficial effects on electrolyte balance and recovery. As acai is high in sodium and potassium, consumption of acai juice may promote restoration of fluid electrolyte balance after exercise. Furthermore, the antioxidant properties of acai juice have been associated with muscular recovery [14]. During exercise, reactive oxygen species (ROS) and free radicals that accumulate may result in exercise-induced tissue damage. Polyphenolic compounds found in acai that exhibit antioxidant abilities can be a good source of exogenous antioxidants. In combination with endogenous antioxidants, these exogenous antioxidants can help protect against radical-mediated oxidative stress as a result of exercise [18]. Existing research has associated acai consumption with reduced oxidative and muscular stress [19]. Concurrently, the high protein content in acai can aid in muscle recovery after exercise. While promising, whether acai juice is a useful recovery drink needs further investigation.

In contrast, the effect of consuming acai on exercise performance is uncertain. While acai consumption improved anaerobic tolerance and aerobic capacity in male cyclists [20], consumption of an acai berry-based juice blend did not improve sprint performance in junior athletes [19]. The limited research in this area makes the effect of acai consumption on performance unclear. This may be because most of the carbohydrates found in acai are dietary fibers [15]. Unlike simple sugars such as glucose and fructose, dietary fibers are digested less readily. Thus, performance-enhancing effects of acai consumption might be less measurable than its recovery effects.

It is noteworthy that pure acai juice is often mixed with other berries and fruits when sold commercially to improve its taste. Its nutritional value therefore will vary among different mixtures. Additional research is required to determine the nutritional content of acai when prepared differently. Moreover, anthocyanin levels in clarified acai juice are found to be less than in acai pulp [21]. A proposed solution to counter this is to free up more anthocyanin by breaking down the insoluble fiber in the acai pulp during its juice preparation [21]. These technical modifications can amplify the antioxidant properties in acai juice.

Taken together, the high sodium and potassium content in acai may help replenish electrolytes lost during exercise. The high protein content and presence of polyphenolic compounds render acai juice a good drink to promote muscular recovery after physical activities. However, as acai is typically not consumed on its own, the effects of acai on athletes may be less remarkable when consumed in commercially available forms, such as acai-based juice blends. Nevertheless, given its popularity, it may be worthwhile to investigate how often acai is consumed after exercise in Brazil, along with the effects of acai consumption on hydration status.

### 2.2. Sugarcane juice

Sugarcane (*Saccharum officinarum*) is native to tropical countries such as Brazil and India, which together produce more than half of the world’s sugarcane [22]. In India, it is suggested that sugarcane juice is a common beverage among athletes [6]. Freshly squeezed sugarcane juice can be prepared easily from sugarcanes by extracting its juice using a machine. Freshly squeezed sugarcane juice is rich in proteins, phytonutrients, and minerals [23].

During exercise, consumption of sugarcane juice has demonstrated similar effects on rehydration as a typical sports drink [6]. Furthermore, ingestion of sugarcane juice resulted in a greater increase in blood glucose concentrations than both the sports drink and plain water during the post-exercise recovery phase. Additionally, sugarcane juice can improve carbohydrate uptake. While majority of the carbohydrates in sports drinks are glucose and its polymers [24], sugarcane juice is high in sucrose, with lesser amounts of glucose and fructose [25]. The presence of fructose and sucrose, a glucose-fructose dissacharide, may improve the effectiveness of carbohydrate absorption due to the involvement of a separate fructose uptake mechanism which will not interfere with the glucose uptake mechanism [24].

Sugarcane juice may also be a good source of carbohydrates to augment recovery. Drinking sweetened beverages can increase voluntary fluid ingestion during exercise in hot environments [26]. This may encourage athletes to consume more, which increases the chances of meeting individual hydration requirements. Concurrently, simple sugars from sugarcane juice that are absorbed by the body can replenish glycogen storage, which is usually depleted after high-intensity exercise. Similar to acai juice, antioxidants from phytonutrients in sugarcane juice can help to reduce oxidative stress accumulated during exercise [18]. Overall, this can promote recovery in a holistic manner through rehydration, restoration of glycogen storage and promotion of muscular recovery.

However, consumption of freshly squeezed sugarcane juice can reduce gastric emptying. Based on ACSM recommendations, carbohydrate intake during exercise will not compromise fluid replacement if the carbohydrate content of the beverages is less than ~8% [2], whereas freshly squeezed sugarcane juice contains 9% carbohydrate [6]. High carbohydrate concentration reduces gastric emptying rate [2] and can impair fluid absorption during exercise [26], potentially resulting in an undesired deterioration in exercise performance [26]. Although trained athletes may be able to tolerate higher intake of carbohydrates [27], untrained athletes might consider diluting freshly squeezed sugarcane juice to ensure carbohydrate concentration is within recommended range. Another option would be to use sugarcane juice as a supplement instead of the main source of rehydration by incorporating it into post-exercise meals.

Future research should determine the nutrient profile of sugarcane juice including the levels of antioxidants, as this would allow athletes to improve their understanding of the benefits of drinking sugarcane juice. Additionally, high sucrose concentrations may lead to adverse gastrointestinal symptoms due to fructose malabsorption [28]. A study found that fructose was malabsorbed in 6 out of 16 participants when sucrose was ingested [29]. Moreover, fructose malabsorption was associated with cramps and/or diarrhea in five out of six participants [29]. Therefore, the practicality of sugarcane juice as a fluid replacement strategy warrants further investigation.

Sugarcane juice may be a useful beverage for athletes during and after exercise, especially in tropical countries where it is culturally popular. Future studies can investigate the extent to which sugarcane juice is consumed after exercise in these countries. Adjustments should be made to the nutritional content of freshly squeezed sugarcane juice to ensure the carbohydrate concentration is within recommended levels. While Kalpana et al. [6] have shown similar effects of consuming sports drinks and sugarcane juice on rehydration during exercise, further investigations can test different indices of exercise performance and recovery, or include other types of sports apart from endurance-based sports in order to expand the current literature on the effects of sugarcane juice on hydration.

### 2.3. Beso

In the Horn of Africa, Ethiopian athletes are well-known for their prowess in long distance endurance events. In a study on the athletes’ culture, it was revealed that a popular drink among Ethiopian athletes is *beso*, a roasted barley drink [7]. *Beso* is prepared by adding either hot or cold water to barley flour. Water is added until the mixture can be rolled by hand. Salt, sugar, or melted spiced butter may also be added to the mixture. Additionally, the flour can be mixed with cold water and sugar and served as a beverage [8]. *Beso* can be drunk immediately or with the addition of honey.

Regarding exercise performance, *beso* might not be a good drink to consume during exercise as it is low in simple sugars. While carbohydrate consumption at a rate of 30–60 g·h^−1^ is recommended to sustain performance [2], the majority of carbohydrates found in roasted barley are dietary fibers and starch [30]. Since dietary fibers and starch are less resistant to digestion and absorption compared to simple sugars, consuming *beso* during exercise may be ineffective in improving performance.

Currently, there are no studies detailing the effect of *beso* on athletic performance or recovery. Little is also known about the exact nutritional content of *beso*. The nutritional content of roasted barley suggests that *beso* may be a good drink to consume after exercise for promoting recovery. Barley is high in phenolic compounds, which display antioxidant activities. Roasting of barley results in a significant increase in these antioxidant activities [31]. Antioxidant intake from foods and beverages can help to lower production of ROS, which may reduce the likelihood of exercise-induced tissue injury [19].

There is very limited understanding of the effects of *beso* on rehydration, performance, and recovery. It will be useful to determine the exact nutritional content of *beso* before further studies investigate its effects, if any, on rehydration, exercise performance, and recovery.

### 2.4. Beer

Beer is made from water, malted barley, hops, and yeast. Most full-strength beers contain about 5.0% alcohol by volume (ABV) while low-strength beers contain approximately 2.5% ABV. Usually consumed after amateur sports for social reasons [32], beer is believed to aid in hydration as it is 90% water [10].

However, the alcohol in beer can worsen dehydration via excess urine production [1]. Additionally, beer is low in sodium [10] which is not helpful in replenishing sodium lost through sweat during exercise. Therefore, NATA’s Position Stand discourages consumption of drinks with >4% alcohol after exercise as it can decrease performance and recovery rate [1]. Existing research on the effects of beer consumption on exercise performance, thermoregulation, and hydration produced contradictory findings [33,34]. This can be expected as these studies vary in study designs [34]. Another possible reason for the discrepant findings is the differing levels of alcohol tolerance between individuals in the studies. In humans, the enzymes involved in alcohol metabolism are alcohol dehydrogenase and aldehyde dehydrogenase. Inter-individual genetic variations in alcohol dehydrogenase and aldehyde dehydrogenase allelic expressions are associated with differences in alcohol tolerance [35]. However, studies exploring the effects of beer consumption in athletes do not account for the differences in alcohol tolerance among individuals, which makes it difficult to set thresholds between low and high alcohol intake [36]. Since greater alcohol tolerance can reduce the physiological response [37], negative consequences of full-strength beer consumption on performance, thermoregulation, and hydration may be masked in individuals with better alcohol tolerance.

To ameliorate the health risks of alcohol from beer consumption, low-strength, and nonalcoholic beer have been popularized as a sports drink [10]. This is supported by a study showing that nonalcoholic beer can potentially be more effective in maintaining fluid-electrolyte balance than full-strength beer and even water [38]. In combination with the electrolytes present in beer, it is shown that beers containing ≤ 4% ABV do not induce dehydration in well-hydrated individuals [1]. Furthermore, it has been demonstrated that low-strength beer with added sodium can improve fluid balance and reduce urine output after exercise when compared with full-strength beer [39]. However, it is important to note that in these studies, fluid balance was not fully restored regardless of the modifications made to the beers [38,39]. Therefore, while modified low-strength beer might prove to be a healthier alternative to full-strength beer, it might still not be the most desirable post-workout beverage.

Apart from low and nonalcoholic beers, another form of alcoholic sports beverage is sports beer. Table 1 provides information on some sports beers available in the market (Table 1). While manufacturers claim that sports beers are infused with more electrolytes than regular beer, there is a lack of information on the nutritional content of sports beers. To date, no study has investigated the effects of sports beers on hydration. We postulate that sports beers may be beneficial for hydration to a moderate extent as the greater amount of sodium can help restore fluid-electrolyte balance more effectively. However, consumers should be cautious of the high ABV in most sports beers (Table 1), as the higher ABV can increase urine output [1], thereby negating the rehydration effects of the sports beers. Thus, we expect that achieving optimal fluid-electrolyte balance with sports beer would be more difficult than with low-strength beer.

**Table 1.** List of sports beers currently in the market with their respective alcohol by volume (ABV) [40–46]

Beer	Brewery	ABV(%)
FKT	Sufferfest	5.5
Go Play IPA	Avery	5.5
Race Pace	ZeLus	3.7
Rec. League	Harpoon	4.0
Sea Quench Ale	Dogfish Head	4.9
War Llama	Mispillion River	5.0
Quencher	Fifty West	4.5

Future studies are necessary to determine how often and how much beer is usually consumed after exercise. Further investigations are also required to analyze the nutritional content of sports beer and its physiological effects. In a study which investigated the effects of a moderate intake of beer on rehydration, consumption of up to 660 ml of full-strength beer (4.5% ABV) before *ad libitum* water intake did not adversely affect indices of hydration status or the recovery process after exercise [32]. Subsequent intake of water may have diluted the original concentration of alcohol from the beer, thus attenuating the diuretic effect of alcohol consumption. Therefore, it might also be worthwhile to investigate the effects of drinking sports beer followed by *ad libitum* water ingestion.

Low-strength beer may be the best hydration option among full-strength, low-strength, and sports beer after exercise. Given the high prevalence of beer consumption after sports in Western cultures, it is paramount for athletes across all levels to be educated on the effects of alcohol ingestion before and after exercise. Despite alternatives or modifications made to full-strength beer, the deleterious health effects of beer consumption should not be ignored. Intake of low-strength beer and sports beer should also be moderated in order to reap its maximum benefits.

### 2.5. Non-colored sports drink

Non-colored cola, coffee, tea, and beer are gaining popularity in Japan. Apart from allowing workers to boost professionalism at their workplaces, these transparent beverages are reported to be healthier as they are perceived to contain fewer calories [47]. The transparency of these beverages may also attract health-conscious consumers trying to avoid sweetened drinks. This could be due to the positive association between the increasing color of a beverage and consumers’ perception of sweetness [48]. Moreover, existing concerns about artificial coloring in foods have resulted in some consumers seeking coloring-free alternatives [49]. These reasons provide possible theories in explaining the popularity of transparent beverages in Japan.

In the sports drinks market, a similar trend is also observed as non-colored sports drinks manufactured in Japan are gaining popularity. Usually consumed during and after exercise, these sports drinks are appropriately equipped with carbohydrates and electrolytes. Carbohydrates in sports drinks can prolong exercise endurance while electrolytes can aid in post-exercise recovery [1,2]. This unique Japanese culture may be due to the assumption that these beverages are perceived to be healthier than colored beverages. While sports drinks are formulated to aid in electrolyte replacement, it is not known if non-colored sports drinks are indeed healthier than its colored counterparts. Similarly, the reason for its popularity in Japan has also not been investigated.

More research is definitely required to establish if colors present in sports drinks necessarily mean they are less healthy than non-colored sports drinks. Given its popularity in Japan, future studies can also look into the long-term impacts of the daily consumption of these non-colored sports drinks on hydration, exercise performance, recovery, and also health.

### 2.6. Ramadan fasting

Ramadan fasting occurs during the month of Ramadan, where Muslims abstain from food and fluid intake, smoking, and sexual interaction from sunrise to sunset [11]. As the Islamic calendar follows a lunar calendar, the time of year of Ramadan differs yearly. The duration also differs between countries, depending on geographical location and season of the year [11]. While all Muslims are encouraged to fast, exceptions are made to several groups of Muslims. These include those who are pre-pubescent, traveling or unsettled, suffering from illness, in extreme pain from injury, breastfeeding, or pregnant [11].

The restriction on fluid intake during Ramadan fasting can have a greater impact on individuals who exercise due to their increased sweat loss from a greater exercise intensity. This is even more critical when the time of competition does not take into account religious practices during Ramadan. One example is the 2012 Summer Olympics held in London, which partially coincided with Ramadan. Although some sports governing bodies do alter their competition schedule to cater to fasting athletes, such as delaying kickoff time of football matches in Muslim countries until after sunset, this might not always be the case in non-Muslim countries or if Muslim athletes do not make up majority of the athlete population. This results in Muslim athletes being less hydrated than their non-fasting counterparts, which may impair their performance and recovery.

Trabelsi et al. [50] summarized the research findings on the effects of fasting on physiological and biochemical markers. Inconsistent conclusions about hydration status from these findings could stem from the different participant demographics, climate, and hydration assessment technique employed. The contradicting results might also be due to the different individual adaptations to fasting. Nonetheless, Ramadan fasting can lead to athletes starting their exercise in a state of hypohydration [51], which may worsen if exercise is conducted in the later part of the day.

With knowledge of the adverse effects of hypohydration on hydration status, there may be more implications for fasting Muslim athletes as they are unable to hydrate before and during exercise. They are also unable to rehydrate immediately after exercise unless their exercise ends right before fast is broken. It is therefore difficult for fasting athletes to abide by the recommendations from ACSM [2] and NATA [1]. Given that the body also receives fluids via food moisture content, the lack of fluid intake during Ramadan is further compounded by restrictions on food intake throughout the day.

Research on the effects of Ramadan fasting on exercise performance output produced varying results [52]. A possible reason is the different factors involved in the maintenance of performance mechanisms apart from dehydration, for example sleep quality. However, this small margin in disparity is imperative as it may be the difference between first and second place in a competitive sports setting. As a result, athletes need to adopt proper hydration practices, along with meeting their individual requirements of other physiological and mental indices, in order to maximize their performance output while celebrating Ramadan throughout the month.

Fasting individuals should ensure sufficient hydration during their pre-dawn meal and be conscious of the amount of fluid intake when breaking fast. Fasting athletes may have a tendency to consume more water after enduring several hours of dehydration. When managed properly, negative consequences of hypohydration can be prevented, allowing athletes to train or compete safely.

### 2.7. Avoiding cold water during and after exercise in Chinese culture

According to Chinese culture, it is recommended that food and drinks should be consumed at room temperature. Based on Traditional Chinese Medicine (TCM), it is important for the body to maintain its “hot-cold” balance. TCM advises against ingesting cold food and drinks during warm weather because it can increase dampness in the body and irritate the gastrointestinal tract, causing stomach and intestinal cramps [53]. Moreover, it is believed that cold food and drinks can place unnecessary stress on the digestive system as more energy is required to raise the temperature of the food [4,53].

The preference for warm drinks over cold drinks extends even to hydration during and after exercise. According to TCM, consumption of warm water over cold water after exercise is advised. Anecdotally, consumption of cold water can ‘shock’ internal organs after body temperature rises during exercise, and does not facilitate natural cooling of the body after exercise [4]. This explains why cold water is avoided in Chinese culture.

Conversely, ingesting water at cooler temperatures is recommended by position stands as the better fluid palatability can improve fluid replacement [1,2]. As noted by Tan and Lee [54], these recommendations on consuming water at cooler temperatures are based on studies demonstrating greater *ad libitum* fluid replacement.

Tan and Lee [54] summarized the physiological effects of consuming water at different temperatures at rest and during endurance exercise. Studies have shown that consumption of cold water can lower core body temperature and attenuate the effects of heat strain. However, this effect was noted to be less prominent when fluid was ingested during exercise due to the resultant body’s natural thermoregulatory response to heat debt. It may thus be useful for cold water to be consumed before exercise as a form of pre-cooling.

Drinking cold water has also been shown to reduce heat stress effects more effectively than drinking warm water after exercise. When water at 4°C was ingested after exercise, there was a greater decrease in body core temperature than when water at 28°C was ingested [55]. In the same study, skin temperature and heart rate were also lower when cold water was ingested than when warm water was ingested [55]. This reduction in body core temperature can also help to extend exercise duration before hyperthermia and thus improve endurance performance [55].

TCM literature however does not support cold water ingestion after exercise. There is not only an uncertainty around the negative health impacts that cold water ingestion is believed to have, but there is also no concrete evidence that warm water ingestion after exercise is more beneficial than cold water ingestion for performance or recovery.

As mentioned in ACSM’s position stand, preferred water temperature is subjected to cultural influences [2]. As there are no reports of health implications of drinking cold water after exercise, cold water may not be necessarily harmful toward the body as suggested by TCM beliefs. It is more important that individuals are adequately hydrated after exercise to avoid the adverse effects of hypohydration.

## 3. Practical applications

Individuals need to understand the impacts of their individual behaviors and preferences surrounding hydration on their exercise performance and recovery in order to make an informed choice. While cultural forces can deviate hydration practices from current evidence-based fluid replacement recommendations, individuals should still abide by the guidelines provided by professionals to ensure proper fluid replacement. Drinks that are inconsistent with current evidence-based fluid replacement recommendations should be best avoided after exercise. As it is difficult to alter hydration practices that have been embedded in the various cultures, modifications can be made to some of these culturally popular drinks based on evidence for inclusion in the athlete’s hydration plans. It is important to be aware of the actual nutritional content of these culturally popular beverages, rather than relying on anecdotal evidence of their nutritional profile to avoid adverse effects (Table 2).

**Table 2.** Country of origin and nutritional content of various culturally popular drinks [6,40,56–59]

Type of Beverage (Amount)	Country of Origin	Energy (kCal)	Carbohydrate (g)	Protein (g)	Fat (g)	Sodium (mg)	Potassium (mg)	Calcium (mg)	Magnesium (mg)	Other Ingredients and Nutrients
Heineken Beer (100 ml)	The Netherlands	42	3.2	0	0	-	-	-	-	-
Sufferfest FKT (12 fl oz)	U.S.A.	175	14	1.7	0	58	-	18	-	Blackcurrant, salt
Pocari Sweat (100 ml)	Japan	25	6.2	0	-	20	20	2	0.6	Vitamin C
Suntory Green Da-Ka-Ra (100 ml)	Japan	18	4.4	0	0	-	1-10	0.1-1.0	0.1-1.0	Iron
Sambazon Acai Juice (8 fl oz)	Brazil	140	29	40	3	40	56	31	-	Vitamins A and D, iron, fiber
Sugarcane Juice (100 g)	India, Brazil, and other tropical countries	39	9.1	-	-	20	360	74	-	Antioxidants, vitamins A, B and C, iron, zinc

*There are no known ingredient lists for *beso.*

## 4. Conclusions

Due to cultural forces, it is difficult to ensure that every individual will consume drinks that contain recommended nutrient levels for hydration. Individual preferences, availability of a certain type of drink and religious beliefs can all play an important role in dictating varying hydration practices, which may or may not be in accordance with current evidence-based position stands meted out by professionals. Due to the lack of literature, the effects of such cultural hydration practices are not well-established. Moving forward, it may be worth investigating the impacts of different cultural hydration practices on exercise performance and recovery. This can help consumers understand both the positive and negative implications of their hydration practices, so that they can make informed choices for their hydration practices.
